# Music Therapy Outcomes in Older Adults Using Cochlear Implants, Hearing Aids, or Combined Bimodal Devices: A Systematic Review

**DOI:** 10.3390/healthcare13151795

**Published:** 2025-07-24

**Authors:** Liviu Lucian Padurean, Horatiu Eugen Ștefanescu, Calin Muntean, Vasile Gaborean, Ioana Delia Horhat

**Affiliations:** 1 Doctoral School, “Victor Babeș” University of Medicine and Pharmacy, Eftimie Murgu Square 2, 300041 Timișoara, Romania; lucian.padurean@umft.ro; 2 Municipal Clinical Hospital, Pricazului Str. 16, 335700 Orăștie, Romania; 3 Discipline of Otorhinolaryngology, Department IX, “Victor Babeș” University of Medicine and Pharmacy, Eftimie Murgu Square 2, 300041 Timișoara, Romania; deliahorhat@yahoo.com; 4 Medical Informatics and Biostatistics, Departament III-Functional Sciences, “Victor Babeș” University of Medicine and Pharmacy, Eftimie Murgu Square 2, 300041 Timișoara, Romania; 5 Thoracic Surgery Research Center, “Victor Babeş” University of Medicine and Pharmacy Timişoara, Eftimie Murgu Square No. 2, 300041 Timişoara, Romania; vasile.gaborean@umft.ro; 6 Department of Surgical Semiology, Faculty of Medicine, “Victor Babeş” University of Medicine and Pharmacy Timişoara, Eftimie Murgu Square No. 2, 300041 Timişoara, Romania

**Keywords:** cochlear implant, hearing aid, music therapy, elderly, rehabilitation, quality of life

## Abstract

**Background/Objectives:** Cochlear implants (CIs) and hearing aids (HAs) have enhanced auditory rehabilitation in elderly individuals, yet limitations in musical perception and psychosocial integration persist. This systematic review aimed to evaluate the effects of music therapy (MT) on the quality of life (QoL), self-esteem, auditory perception, and cognition in older CI and HA users. **Methods:** A comprehensive search of PubMed was conducted up to March 2022 following PRISMA guidelines. Studies involving participants aged ≥ 60 years with CIs and/or HAs were included. Ten studies (*n* = 21,632) met eligibility criteria. Data were extracted and assessed using the Newcastle–Ottawa Scale. **Results:** MT led to improved sound quality, with HISQUI19 scores rising from 60.0 ± 21.8 to 74.2 ± 27.5. Early MT exposure was associated with significantly better MUMU outcomes (*p* = 0.02). Bilateral CI users showed enhanced stereo detection (52% to 86%), and CI + HA users achieved CNC scores exceeding 95%. Postlingual CI users outperformed prelingual peers in musical discrimination (9.81 vs. 3.48; *p* < 0.001). Long-term HA use was linked to better a QoL and reduced loneliness. **Conclusions:** While music therapy appears to support auditory and psychosocial functioning in hearing-impaired older adults, the absence of randomized controlled trials limits causal inference regarding its effects. These results support its integration into hearing rehabilitation strategies for older adults.

## 1. Introduction

The cochlear implant (CI) represents a well-established method of auditory rehabilitation in postlingually deafened adults [1]. Modern auditory prostheses significantly enhance speech perception and intelligibility. However, users of both cochlear implants and hearing aids (HAs) require continuous training and rehabilitation in order to achieve music enjoyment and broader auditory satisfaction. In Romania, the prevalence of severe hearing loss was estimated at 1.43% in 2018, with projections indicating a rise to 2.56% by 2050 [2].

Hearing loss predominantly affects the elderly population. Epidemiological studies have consistently shown a growing prevalence of hearing impairment across various demographics, reinforcing the need for focused research and improved rehabilitation strategies. The quality of life (QoL) of individuals with cochlear implants or HAs is of paramount importance. Sensorineural hearing loss, the most common type, impacts approximately 5% of the global population [3,4,5,6]. Music therapy (MT) has been shown to enhance both auditory performance and the quality of life, contributing also to improvements in self-esteem following structured sessions [7,8,9,10].

Beyond auditory function, hearing impairment is associated with diminished psychological well-being, reduced self-esteem, and deteriorated interpersonal relationships. Despite the clinical relevance, current literature remains limited regarding auditory rehabilitation through music therapy in elderly CI and HA users. In older adults, cortical plasticity plays a crucial role in the outcomes of auditory rehabilitation [11,12,13]. Two main strategies exist to improve musical perception in CI users: (1) optimizing device technology, and (2) enhancing perceptual skills. Among the reported benefits of MT, the most prominent is the enhancement of self-esteem [14,15].

Unilateral cochlear implant users with residual hearing in the contralateral ear are an increasingly observed clinical group. Music therapy has gained importance not only for its auditory benefits, but also due to its role in cognitive stimulation, reduction in anxiety and depression, and support for social reintegration. MT sessions typically include guided listening to music—selected to be slow, clear, and familiar—adapted to implant acoustics. These sessions may involve recognition of instruments and vocal timbre, rhythm imitation, singing, and melodic reproduction [16].

The aim of this systematic review was to synthesize available evidence on the impact of music therapy on musical perception, quality of life, and self-esteem in elderly individuals using cochlear implants or hearing aids.

## 2. Materials and Methods

### 2.1. The Protocol and Registration

This systematic review was conducted in accordance with the PRISMA guidelines (Preferred Reporting Items for Systematic Reviews and Meta-Analyses) [17], ensuring a structured, transparent, and reproducible methodology. In January 2025, a thorough and methodical search was carried out in the PubMed, Scopus, and Web of Science electronic database to identify relevant literature published from 2014 to March 2024. The focus of the search was to gather studies addressing the role of music therapy in elderly users of cochlear implants and hearing aids.

The search strategy was built using a combination of Medical Subject Headings (MeSH) and free-text terms, with Boolean operators applied to refine the results. The keywords included the following: “cochlear implant,” “hearing aid,” “music therapy,” “HISQUI19,” “elderly,” “rehabilitation,” “quality of life,” “music training,” “music perception,” “auditory benefit,” “MUMU,” and “self-esteem.” Additional terms such as “perceptive auditive,” “QoL,” and “psychological well-being” were also explored. Grey literature (e.g., conference abstracts) was excluded to ensure methodological rigor. Although no formal language restrictions were applied initially, only English-language articles were ultimately included due to limited translation resources.

We aimed to include original studies such as prospective cohort studies, cross-sectional analyses, and pilot trials, provided they assessed outcomes related to auditory rehabilitation, musical perception, or psychosocial metrics in elderly CI/HA users. To ensure transparency and accessibility, the review protocol was registered with the Open Science Framework (OSF) under the registration code osf.io/d297e. Following screening and eligibility assessment, ten studies meeting the inclusion criteria were selected for full analysis.

This comprehensive and inclusive search strategy was designed to ensure coverage of all relevant evidence, enabling a nuanced understanding of the therapeutic impact of music therapy in older adults with hearing implants. This approach ensures that the review captures the full scope of current knowledge on this topic while maintaining methodological integrity and reproducibility.

### 2.2. Eligibility Criteria

Eligibility criteria were established to identify studies that provide relevant data on the effects of music therapy in elderly users of cochlear implants and hearing aids, particularly in relation to the quality of life, self-esteem, and social reintegration.

The inclusion criteria for this systematic review were as follows:

Study population: Only studies including participants with a mean age over 60 years who used cochlear implants and/or hearing aids were eligible.
Focus of the study: Included research had to specifically assess the impact of music therapy on the quality of life and self-esteem in older adults with hearing loss. This encompassed studies evaluating variables such as auditory rehabilitation, auditory benefit, musical training, auditory and musical perception, and psychological outcomes.
Study design: Eligible designs included prospective studies, cross-sectional studies, longitudinal studies, and pilot studies.
Outcome measures: Studies had to report validated instruments or clearly defined parameters assessing the quality of life, self-esteem, musical perception, auditory perception, or auditory benefit.
Language: Only articles published in English were included.

The exclusion criteria were as follows:

Studies not conducted on human subjects;
Studies lacking specific results or outcome data;
Grey literature, such as non-peer-reviewed articles, conference abstracts, editorials, and general commentaries, which were excluded to ensure methodological rigor and data credibility.

These criteria were defined to ensure that the selected studies provide clinically relevant, well-structured, and measurable insights into the role of music therapy in the auditory and psychosocial rehabilitation of elderly patients with hearing loss.

### 2.3. Definitions

In this systematic review, the term elderly refers to individuals over 60 years of age who use either cochlear implants or hearing aids. This age threshold is consistent with current research trends and clinical observations, which highlight distinct epidemiological patterns and auditory rehabilitation outcomes when comparing younger and older adults with hearing loss.

The QoL in cochlear implant and hearing aid users was evaluated using standardized questionnaires, designed to reliably and sensitively quantify multiple dimensions of daily functioning and well-being. In addition to these instruments, qualitative patient feedback was also considered. Such input provided valuable insight into the subjective and personal aspects of life with hearing loss, i.e., dimensions that are often underrepresented in structured clinical assessments.

### 2.4. Data Collection Process

Data collection was carried out independently by two reviewers, each following the predefined inclusion and exclusion criteria. This dual-review process ensured methodological consistency and minimized selection bias, allowing for only studies specifically addressing music therapy in elderly cochlear implant and hearing aid users to be included.

Disagreements between reviewers were resolved through discussion and, if necessary, consultation with a third party. After screening titles, abstracts, and full texts, 10 studies met all eligibility criteria and were included in the final analysis.

**Figure 1:**
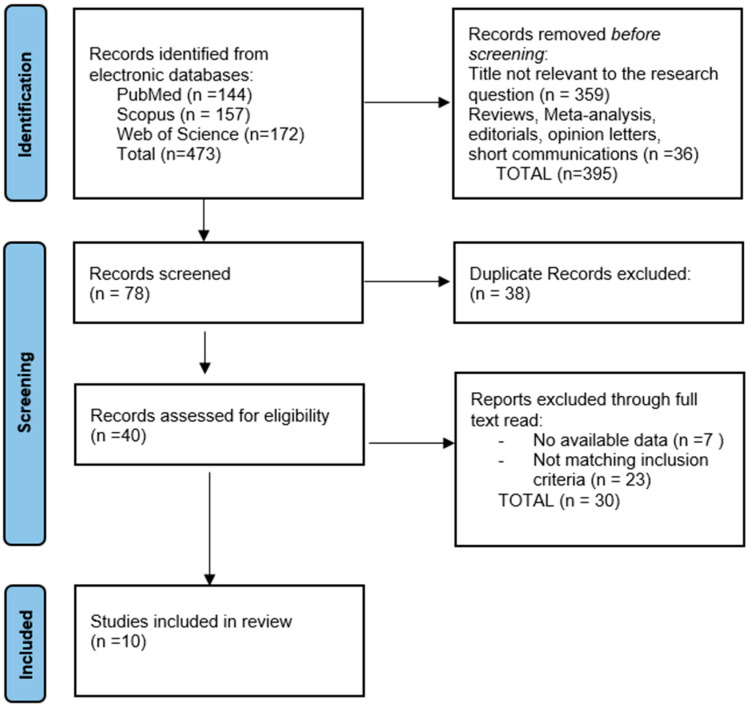
PRISMA flowchart.

This rigorous selection process was designed to ensure the inclusion of high-quality studies, enabling a focused synthesis of the current evidence regarding the impact of music therapy in this patient population. The study selection flow is illustrated in Figure 1, following the PRISMA 2020 flow diagram.

### 2.5. Risk of Bias and Quality Assessment

The quality of the included studies was assessed using the Newcastle–Ottawa Scale (NOS) [18], which evaluates three key domains: (1) selection of study groups, (2) comparability of the groups, and (3) ascertainment of exposure for case–control and cohort designs. Each study received a score reflecting its methodological rigor, which was used to classify the overall quality as low, moderate, or high. As no randomized controlled trials (RCTs) were identified, tools specific to RCTs (e.g., the Cochrane Risk of Bias tool) were not applied.

Two reviewers independently evaluated the included studies using this scale. Discrepancies in scoring were resolved through discussion, and when necessary, in consultation with a third reviewer. This assessment ensured a consistent and systematic identification of studies that met high methodological standards and were appropriate for inclusion in the final synthesis.

## 3. Results

### 3.1. Study Characteristics

The systematic review includes 10 studies [19,20,21,22,23,24,25,26,27,28] presented in Table 1. These studies were conducted in various countries: Austria [19,21], Germany [20,27,28], USA [22,24], Canada [23], Australia [25], and the Netherlands [26]. The research methodology used in these studies included both cross-sectional and prospective cohort studies. Amann et al. [21] and Mick et al. [23] used a cross-sectional approach to gain an overview of the impact of music therapy on the QoL in elderly patients with cochlear implants. Magele et al. [19] and Neuman et al. [24] used prospective cohort studies providing information on the evolution of the impact of music therapy over time. Hutter et al. [20] used a pilot study. These studies were conducted between 2014 and 2024.

### 3.2. Study Characteristics and Design Overview

The ten included studies comprised a total of 21,632 participants, with study designs ranging from small pilot samples to large-scale epidemiological cohorts. Among the ten studies included in the review, six employed a prospective design [21,22,25,26,27,28], whereas the remaining four were retrospective [19,20,23,24].

#### 3.2.1. Intervention Type and Participation

Several studies evaluated the effects of music therapy (MT), either in general form or as targeted musical training. Magele et al. [19] reported that 57% of eligible patients consented to MT, although only 11 completed the intervention. HISQUI scores improved post-MT (from 60.0 ± 21.8 to 74.2 ± 27.5), yet auditory rehabilitation metrics did not reach statistical significance. Participants with a longer CI experience appeared to benefit more.

Fuller et al. [26] compared generic MT to focused timbre/tonality training, with the latter yielding significant gains in both auditory perception and social functioning. Four participants in this group used bimodal configurations.

Hutter et al. [20] included 12 unilateral CI users and evaluated post-implant musical perception following MT, while Amann et al. [21] used a cross-sectional approach in 70 participants to examine subjective hearing quality using HISQUI19.

#### 3.2.2. Device Configuration and Performance

Several studies compared outcomes based on hearing configuration. In the study of Peterson et al. [22], bimodal users (CI + HA) outperformed CI-only users in musical perception. Similarly, Neuman et al. [24] observed significant improvements in low-frequency hearing (LF-PTA reduced from 61.25 dB to 40 dB), and CNC scores exceeding 95% in some CI + HA users. Notably, 18% of participants discontinued HA use, whereas those who maintained it showed better speech and auditory performance.

Buechner et al. [28] reported enhanced stereo detection in bilateral CI users (52% to 86% with direct input), while 57% of the participants accepted MT.

#### 3.2.3. Large-Scale and Neurophysiological Assessments

Mick et al. [23] contributed broad epidemiological data from over 21,000 individuals. Though not focused solely on MT, this enriched the analysis of auditory support use and social participation.

Sarant et al. [25] highlighted the benefits of long-term HA use—particularly after 19 months—on QoL outcomes.

Bruns et al. [27] used event-related potential (ERP) analysis to compare auditory semantic processing. Postlingual CI users demonstrated N400 responses similar to normal-hearing individuals, while prelingual users lacked this neural signature.

##### Musical Perception: Tone, Rhythm, Timbre, and Stereo

Buechner et al. [28] reported a substantial improvement in stereo detection among bilateral cochlear implant users. Performance increased from 52% in free-field conditions to 86% when direct audio input was provided. In contrast, participants with bimodal configurations (CI + HA) were unable to detect stereo cues reliably.

Bruns et al. [27] investigated semantic processing of music using event-related potentials (ERPs). The postlingual CI group exhibited a measurable N400 effect, indicating the ability to assign meaning to musical input. This effect was absent in the prelingual CI group. The mean time interval between the initial speech processor fitting and EEG recording was 18 months (range: 5–103 months), suggesting that extended auditory experience contributes to the development of musical semantic understanding.

Peterson et al. [22] assessed pitch and melody discrimination. The results showed that CI + HAs users performed significantly better than CI-only users, reinforcing the advantage of bimodal stimulation in musical perception. These findings support the recommendation to maintain hearing aid use in the contralateral ear to optimize melodic and tonal discrimination.

Amann et al. [21] found that participants scored lower on the HISQUI19 immediately after cochlear implantation if they were in an older subgroup. This suggests that age may negatively influence subjective sound quality perception post-implant.

Magele et al. [19] compared MUMU scores between groups who received MT before significant hearing loss and those with untreated hearing loss. The MT group demonstrated significantly better outcomes (*p* = 0.02). However, no significant improvements were noted after CI implantation alone (*p* = 0.441), suggesting that early initiation of music therapy may be more effective. Additionally, self-esteem scores were higher post-MT, although motivational scores did not differ significantly between the pre- and post-MT groups.

Across studies, several consistent patterns were observed:

Bimodal users (CI + HAs) achieved better musical perception scores than CI-only users [22];
Postlingual CI users were able to restore semantic musical processing, while prelingual users could not [27];
Bilateral CI users consistently outperformed unilateral users in auditory tasks, particularly those involving stereo detection and spatial sound localization [19].

These results collectively highlight the differential effects of hearing configuration and hearing history on musical perception outcomes.

### 3.3. Disease Characteristics

Table 2 presents detailed comparisons of the disease characteristics across the 10 studies. It shows the diversity in disease duration, severity, surgical history, treatments performed, and complications in cochlear implant patients.

### 3.4. Quality of Life (QoL)

The reviewed studies consistently report improvements in the QoL, self-esteem, and social integration following cochlear implantation, hearing aid use, and MT. These effects were observed across various device configurations and intervention types.

Bimodal users (CI + HA) showed superior outcomes compared to CI-only users. Peterson et al. [22] reported improved sound discrimination and social interaction when a hearing aid was used in the contralateral ear. Similarly, Neuman et al. [24] reported that CI + HA users enjoyed music more and had a significantly better QoL and self-esteem than those who discontinued HA use. This was further supported by Mick et al. [23], who noted that sensory loss—especially vision or dual impairment—was linked to reduced social participation, while those undergoing MT and visual rehabilitation achieved better reintegration.

MT was associated with psychosocial improvements. Magele et al. [19] observed good general health and QoL among CI users, with MT enhancing self-esteem. Hutter et al. [20] and Amann et al. [21] linked improved sound quality (HISQUI19) to better social functioning and subjective well-being.

Fuller et al. [26] found that only targeted tonality/timbre training led to meaningful gains in social reintegration, while general music therapy had limited effects.

Sarant et al. [25] reported reduced loneliness (43.9%) and very low depression rates (4.1%) after 18 months of continuous HA use. These users also demonstrated moderate-to-large QoL improvements as measured by the HUI-3.

Differences based on auditory history were highlighted in Bruns et al. [27], where postlingual CI users performed better in music-based tasks and reported a higher QoL than prelingual users. Buechner et al. [28] additionally reported that stereo detection gains in bilateral CI users were accompanied by improvements in cognition and social reintegration.

Fuller et al. [26] observed significant cognitive improvement only in participants who received tonality/timbre-specific training. This subgroup outperformed both the generic MT and control groups across signal-to-noise ratio (SNR) tests. For example, in the tonality/timbre group, performance at 10 dB SNR reached 16.7 dB, while the generic MT group performed worse (−4.0 dB). At lower SNRs (5 dB and 0 dB), similar patterns persisted, with the tonality group scoring 22.3 and 30.6 dB, respectively, compared to −3.2 and −1.2 dB in the generic group.

Bruns et al. [27] highlighted significant differences in musical discrimination abilities based on auditory history. Postlingually deafened CI users had significantly better scores (9.81, *p* < 0.001) compared to the prelingual group (3.48, *p* < 0.004), who performed below chance level. These findings support the notion that prior auditory experience enhances musical-cognitive integration post-implantation.

Longer durations of deafness (over 20 years), as reported in Fuller [26], Bruns [27], and Sarant [25], were consistently associated with less favorable post-implant outcomes. This highlights the importance of early intervention in hearing restoration strategies.

Music therapy programs varied in frequency and duration across studies. Most commonly, studies implemented between 6 and 12 guided MT sessions, as in Astrid [19], Hutter [20], and Fuller [26]. In the latter, multiple formats were tested: 2 h/week for 6 weeks, 1 h/week for 6 months, 15 min/day for 4 days, and intensive blocks of 3 h/day for 5 days. Only the structured tonality/timbre formats were associated with significant gains.

## 4. Discussion

### 4.1. Principal Findings and Interpretation

This systematic review analyzed 10 studies involving 21,632 elderly participants with hearing loss who underwent CI, used HA, or both (CI + HAs), aged 60–85 years. The studies collectively indicate that MT is associated with improvements in musical perception, self-esteem, and QoL, particularly in postlingually deafened adults and users of bilateral CI or bimodal configurations. Buechner et al. [28] reported that stereo detection improved from 52% in free-field conditions to 86% with direct audio input in bilateral CI users following MT, suggesting an enhancement of spatial auditory perception that can contribute to better music appreciation and speech in noise understanding. Magele et al. [19] showed that 10 guided MT sessions resulted in a significant increase in subjective sound quality, with HISQUI19 scores rising from 77.3 to 123.1 (*p* < 0.001). Additionally, this group focused on pitch discrimination and showed significant improvement from a baseline score of 3.2 ± 1.4 to 4.4 ± 1.2 (*p* = 0.020) after MT. Cortical and auditory plasticity are improved by music therapy [29,30]. Auditory working memory and temporal sequencing are stimulated by MT capacity [31,32]. Picou et al. [33] emphasized its role in activating top–down cortical processing, aiding in the reintegration of emotional and semantic information associated with sound.

### 4.2. Differential Impact Based on Implant Type and Hearing History

The effects of MT are modulated by auditory history and hearing device configuration. Studies in this review demonstrate that postlingual hearing loss CI users respond better to MT than CI users with prelingual hearing loss. Event-related potential (ERP) data show that only postlingual users exhibit N400 responses to musical stimuli, indicating preserved semantic–auditory processing. In contrast, prelingual users lack such activation, suggesting reduced cortical engagement with music. This observation is supported by findings from Illg et al. [34], who note that long-term auditory deprivation limits neuroplasticity, thereby reducing the efficacy of interventions like MT in individuals without prior auditory experience. Therefore, MT appears most effective when auditory and linguisitic frameworks have already been established prior to hearing loss [35]. The type of hearing device also plays a significant role. Across the reviewed studies, CI + HA users consistently outperformed CI-only users in musical pitch and melody recognition tasks. For example, bimodal users had significantly higher melody recognition scores (mean = 73%, SD = 7.3%) than those with CI alone (mean = 56%, SD = 11%; *p* = 0.029). In this review, “structured music therapy” refers to interventions that follow a pre-defined sequence of sessions delivered across multiple time points. Drawing on the protocols described by Fuller et al. [26], we considered a minimum of six sessions—with consistent therapeutic content and active facilitation—to represent a reasonable threshold. This distinction allowed us to separate structured therapeutic approaches from informal or one-time music-based activities.

The preservation of low-frequency hearing in HA users likely enhances pitch contour detection and timbral resolution, contributing to a richer musical experience. Age itself is an influencing factor. Elderly CI users (>65 years) exhibit reduced engagement with music post-implant compared to younger users, both in terms of listening duration and emotional response. These findings suggest that MT protocols for older adults must be adapted to their preferences and processing capacity, favoring familiar musical material and slower pacing. Neuman et al. [24] showed that participants who continued HA use after CI surgery reported a better QoL, self-esteem, and communication outcomes than those who discontinued HA use. This aligns with guidelines for bimodal rehabilitation, which recommend continued HA use in the non-implanted ear to optimize binaural integration and support interventions like MT [34,36,37].

### 4.3. Quality of Life and Psychosocial Reintegration

Several studies reviewed reported improvements in the QoL following MT. For instance, one study documented that only 4.1% of participants who received HA and MT reported depressive symptoms after 18 months, indicating a positive impact on emotional resilience. Another reported that CI + HA users had better scores in social integration, QoL, self-esteem, and daily communication than CI-only users, suggesting that auditory richness enhances self-esteem, emotional, and social stability. Peterson et al. [35] highlighted the additional risk of social isolation in elderly users with dual sensory loss (hearing and vision). In such cases, MT may provide both social integration, cognitive stimulation, and a platform for meaningful interaction, helping preserve independence and reduce feelings of loneliness [38,39,40].

### 4.4. Influence of Training Duration and MT Modality

The efficacy of MT is closely tied to its structure and delivery. Magele et al. [19] demonstrated that 10 structured MT sessions resulted in substantial gains in subjective hearing satisfaction, highlighting the importance of program length. Other studies found that task-specific MT focused on timbre, pitch, and vocal reproduction yielded superior results compared to generic music exposure [23]. External studies confirm that extended and interactive MT formats offer the greatest benefits [41]. Park et al. [42] reported significant gains in both auditory and cognitive performance following a 16-session MT program in older adults with mild cognitive impairment. Similarly, other studies showed that interactive MT (e.g., singing, rhythmic exercises) led to better post-intervention music recognition and higher self-reported enjoyment [26,43,44].

Grenier et al. [45] proposed multimodal MT approaches that combine auditory training with motor and cognitive stimulation. Such models may be particularly well suited for older adults with comorbidities, providing a more holistic rehabilitation experience.

Several patient-related and technical factors may critically influence the outcomes of music therapy in individuals with hearing devices. Cognitive status is increasingly recognized as a key moderator of auditory rehabilitation, yet most studies included in this review did not assess it systematically. Notably, Sarant et al. [25] demonstrated cognitive gains following hearing aid use, supporting the role of cognitive plasticity. However, without standardized screening tools such as the MoCA, it remains challenging to isolate the true effect of music-based interventions. Additionally, cochlear implant programming strategies are evolving, and anatomy-based fitting methods, including selective electrode deactivation, have shown promise in enhancing pitch perception and sound quality [46]. These techniques, although not applied in the reviewed studies, could potentiate the effects of music therapy and warrant inclusion in future research protocols. In parallel, advanced signal processing options in hearing aids—such as frequency compression and transposition—may improve access to high-frequency acoustic cues crucial for music perception. Given their potential influence, future studies should report fitting parameters and assess their impact. Another important subgroup often overlooked in analyses is that of EAS users, who benefit from both acoustic and electric hearing. EAS has been associated with superior performance in timbre and melody recognition [47], yet outcomes in this population were not evaluated separately in the included studies. Moreover, CI candidates with retrocochlear pathologies, such as tumor removal, may exhibit limited benefit due to compromised neural integrity. In such cases, electrophysiological assessments like EABR or EALR may guide candidacy decisions, particularly when music enjoyment is a rehabilitative goal. Lastly, individual history of music appreciation may act as a predictor of therapy responsiveness. Lam et al. [48] showed that greater pre-implant musical engagement correlated with improved post-implant chord discrimination and enjoyment. These findings suggest that prior musical experience may enhance neural receptivity to auditory training and should be considered when selecting and stratifying patients in future trials.

### 4.5. Subjective Sound Quality Perception

Subjective assessments of sound quality, such as those measured by HISQUI19 and MUMU, are critical for evaluating the real-world effectiveness of MT. One study included in this review showed that HISQUI19 scores were significantly lower in users over 60 years than in younger adults, even after similar CI use durations. This finding highlights the impact of aging on auditory plasticity and suggests that MT alone may not fully offset age-related limitations. In contrast, another study found that MT initiated prior to the onset of severe hearing loss was associated with significantly higher MUMU scores (*p* = 0.02), while MT applied only after CI implantation did not yield statistically significant benefits (*p* = 0.441). These results reinforce the importance of early intervention in optimizing rehabilitation outcomes. Additionally, subjective evaluations showed that average VAS scores for sound quality improved from 60.0 ± 21.8 to 74.2 ± 27.5 (*p* = 0.021) after MT. These changes were accompanied by increased confidence and enjoyment in music listening, supporting the integration of user-reported outcomes in auditory rehabilitation programs. Creech et al. [46] further emphasized that personalized MT, particularly when supported by accessible technology and adapted for elderly users, increases compliance and long-term benefit. Holder et al. [36] echoed this, recommending that subjective tools such as HISQUI and MUMU be routinely used in clinical practice to guide individualized intervention plans, ideas encouraged by other studies as well [49,50].

### 4.6. Study Limitations

This systematic review is subject to several limitations that may influence the generalizability and interpretation of findings. The first limitation of this review is the high variability in study designs and sample sizes, which ranged from small-scale experimental studies to large epidemiological cohorts. This heterogeneity may limit direct comparability across the findings. While subgroup analyses by study type would be valuable, the small number of studies within each category prevented meaningful stratification. Future reviews with larger evidence bases should consider design-specific subgroup analyses. Second, the lack of randomized controlled trials limited the ability to draw causal inferences about the effects of music therapy (MT). Third, although validated tools such as HISQUI19 and MUMU were employed, subjective reporting biases cannot be excluded, particularly in studies relying on self-reported outcomes without blinding. Another limitation is the lack of consistent reporting on the etiology of hearing loss, which may influence music perception outcomes, particularly in cochlear implant users. Furthermore, the majority of the included studies focused on postlingual cochlear implant users, reducing applicability to prelingually deafened individuals. Language restrictions to English-only publications may have also excluded relevant data from non-English sources. Finally, differences in device type, MT format (general vs. structured), and inconsistent reporting of adherence or long-term outcomes further limit the comparability of results. These limitations underscore the need for future high-quality, longitudinal randomized studies with standardized MT protocols to more precisely determine the role of MT in auditory rehabilitation among elderly CI and HA users.

## 5. Conclusions

This systematic review supports the use of MT as an effective adjunct in the rehabilitation of elderly CI and HA users. MT improves auditory perception—especially rhythm—sound quality, self-esteem, and social reintegration. Structured training formats, such as timbre and tonality exercises, appear more effective than general approaches and may enhance cognitive function. Better outcomes were observed in bimodal and bilateral CI users, while prelingual deafness was associated with limited benefits. Long-term HA use was positively correlated with an improved quality of life and reduced loneliness. While the findings suggest positive trends, the absence of randomized controlled trials limits causal interpretation. Future research should prioritize high-quality experimental designs to establish the efficacy of music therapy in this population.

## Figures and Tables

**Table 1:** Studies included.

Study	Sample Size	Country of Origin	Gender Distribution Women: Men	Comparison Group	Brand of CI/HA
1. Magele et al. [19]	11	Austria	5:6	with MT without MT	CI: Med-EL (OPUS22/Sonnet) Cochlear (CI522/Kanso); HA:NR
2. Hutter et al. [20]	12	Germany	NR	MSCS and MS	NR
3. Amann et al. [21]	70	Austria		IC > 60 years IC < 60 years	NR
4. Peterson et al. [22]	14	USA	NR	IC with CI + HAs	NR
5. Mick et al. [23]	21,241	Canada	NR	SD	NR
6. Neuman et al. [24]	94	USA	NR	IC + HA	CI: Med-EL (OPUS22/Sonnet) Cochlear (N5/N6); HA: Phonak, Oticon, Unitron, Siemens, Telex, Widex, Audibel
7. Sarant et al. [25]	98	Australia	53:45	SD/GML/DET/ONB/IDN/OCL	
8. Fuller et al. [26]	19	Netherlands	NR	CTMTA	CI: CI24RE, HiRes90 K Helix, CI512, CI24R CS, CI24RECA
9. Bruns et al. [27]	53	Germany	21:32	15PreCIU38PostCIU	Med-EL, Cochlear, Advanced Bionics
10. Buechner et al. [28]	20	Germany	NR	Bilateral CI	NR

MSCS = Multidimensional Self-Concept Scales. MS = the musical subtests for melody recognition and for timbre identification in the unilateral condition. SD = sensory deficit is associated with reduced social function. GML = executive function; DET = psychomotor function; ONB = working memory; IDN = visual attention; OCL = visual learning; MMSE = Mini Mental State Examination. PreCIUs = Prelingual CI users. PostCIUs = Postlingual CI users. Bilateral CI users = stereo detection increased from 52% correct in the free field to 86% with direct audio presentation. MT = music therapy; NR = not reported.

**Table 2:** Key comparisons.

Study Number	Duration of Deafness (y)	Onset/Severity of Hearing Loss	Surgical History	Treatment
1. Magele et al. [19]	18, 25 ± 19, 50 years	PH/NR	PMB	CI
2. Hutter et al. [20]	NR	PH/NR	PMU	CI
3. Amann et al. [21]	<20 years; >20 years	PH/NR	PMU	CI
4. Peterson et al. [22]	NR	PH/NR	PMU	CI + HA
5. Mick et al. [23]	45–89 years	NR	NR	NR
6. Neuman et al. [24]	64, 5–64,8 years	NR/Severe/profound hearing loss	PMU	CI + HA
7. Sarant et al. [25]	23 years	NR/Moderate hearing loss	PMB	HA
8. Fuller et al. [26]	35 years	PH/NR	PMB	CI
9. Bruns et al. [27]	31 years	PH/PRH/NR	PMB	CI
10. Buechner et al. [28]	NR	NR	PMB	CI

NR—not reported; PMB—posterior mastoidectomy bilateral; PMU—posterior mastoidectomy unilateral; HAs—hearing aids; CI—cochlear implant; PH = postlingual hearing loss; PRH = prelingual hearing loss.

## Data Availability

Not applicable.
